# Sub-Lethal Effects of Pesticides on the DNA of Soil Organisms as Early Ecotoxicological Biomarkers

**DOI:** 10.3389/fmicb.2020.01892

**Published:** 2020-08-18

**Authors:** Costantino Vischetti, Cristiano Casucci, Arianna De Bernardi, Elga Monaci, Luca Tiano, Fabio Marcheggiani, Maurizio Ciani, Francesca Comitini, Enrica Marini, Eren Taskin, Edoardo Puglisi

**Affiliations:** ^1^Department of Agricultural, Food and Environmental Sciences, Marche Polytechnic University, Ancona, Italy; ^2^Department of Life and Environmental Sciences, Marche Polytechnic University, Ancona, Italy; ^3^Department for Sustainable Food Process, Faculty of Agriculture, Food and Environmental Sciences, Catholic University of Sacred Heart, Piacenza, Italy

**Keywords:** pesticides, DNA, soil microorganisms, earthworms, ecotoxicological biomarkers, denaturing gradient gel electrophoresis, comet assay, next-generation sequencing

## Abstract

This review describes the researches performed in the last years to assess the impact of pesticide sub-lethal doses on soil microorganisms and non-target organisms in agricultural soil ecosystems. The overview was developed through the careful description and a critical analysis of three methodologies based on culture-independent approaches involving DNA extraction and sequencing (denaturing gradient gel electrophoresis, DGGE; next-generation sequencing, NGS) to characterize the microbial population and DNA damage assessment (comet assay) to determine the effect on soil invertebrates. The examination of the related published articles showed a continuous improvement of the possibility to detect the detrimental effect of the pesticides on soil microorganisms and non-target organisms at sub-lethal doses, i.e., doses which have no lethal effect on the organisms. Considering the overall critical discussion on microbial soil monitoring in the function of pesticide treatments, we can confirm the usefulness of PCR-DGGE as a screening technique to assess the genetic diversity of microbial communities. Nowadays, DGGE remains a preliminary technique to highlight rapidly the main differences in microbial community composition, which is able to give further information if coupled with culture-dependent microbiological approaches, while thorough assessments must be gained by high-throughput techniques such as NGS. The comet assay represents an elective technique for assessing genotoxicity in environmental biomonitoring, being mature after decades of implementation and widely used worldwide for its direct, simple, and affordable implementation. Nonetheless, in order to promote the consistency and reliability of results, regulatory bodies should provide guidelines on the optimal use of this tool, strongly indicating the most reliable indicators of DNA damage. This review may help the European Regulation Authority in deriving new ecotoxicological endpoints to be included in the Registration Procedure of new pesticides.

## Introduction

Anthropogenic activities are associated with the massive disposal of contaminants that, in many cases, could be potentially genotoxic and carcinogenic. This poses a major challenge for regulatory authorities and environmental managers to protect the quality and the services provided by natural resources. Simple detection/quantification of xenobiotics in abiotic and biotic compartments has limited relevance, particularly when they occur as complex mixtures, unless their biological or ecological effects are properly evaluated. Biological systems indeed provide important information, which is not readily available from direct chemical analyses of the environmental samples and, thus, are increasingly used as diagnostic tools for integrated environmental management (Sarkar et al., 2006).

The effects of agrochemicals on the soil ecosystem components represented by microorganisms and macroinvertebrates deserve particular attention. The relationship between plant root apparatus and soil microbial communities is strictly linked with soil fertility at a chemical, biochemical, and microbiological level, and several studies on the interactions between root exudates and soil microbial biomass growth, structure, and activity were carried out in detail during the last 20 years (Mergel et al., 1998; Nannipieri et al., 2008; Steinauer et al., 2016; de Vries et al., 2019). Rhizobacteria and fungi can stimulate plant growth through the production of phytostimulators (auxins, gibberellins), increase nutrient uptake, and induce tolerance to plants against abiotic stress or by suppressing biotic stressors like plant diseases or pests (Vryzas, 2016): any change and/or disturbance to the delicate regulation of these interactions can thus result in an impairment of soil fertility.

The use of pesticides against plants pests, weeds, and pathogens was proven to affect the chemical and biological fertility of soils in several cases, including a number of potential adverse effects versus soil microorganisms and/or non-target organisms. During the past, classical studies were performed evidencing the effects of pesticides on the whole soil microbial biomass and/or on the soil biochemical and enzymatic activities (Perucci et al., 2000; Vischetti et al., 2000, 2002; Puglisi et al., 2005, 2012; Nannipieri et al., 2012; Sofo et al., 2012; Suciu et al., 2019), and contrasting results were derived, highlighting the detrimental effect in the major part of the studies but, in some cases, also stimulating effects due to pesticides acting as a carbon source (Puglisi, 2012).

Pesticide ecotoxicology is a relatively new branch of toxicology, being the study of the adverse effect of pesticides versus non-target organisms, including different species living in the ecosystems. Ecotoxicological indexes relative to different commercial pesticides are included in the Dossier for the Registration Procedure. To authorize a pesticide, risk assessment in the EU, the United States of America (USA), and most other Countries requires that the predicted environmental exposure concentration is below a concentration considered safe for non-target organisms (Boivin and Poulsen, 2017). In the EU, in a first tier of risk assessment, this safe concentration is established by the European Food Safety Authority (EFSA) in cooperation with national agencies of the EU member states through a combination of standard toxicity tests, i.e., tests performed with single chemicals and single species under laboratory conditions without additional stressors, and safety factors that account for uncertainties in the extrapolation to real ecosystems [European Parliament, 2009; European Union [EU] (2011)]. The current methodologies to assess the toxicity of pesticides for terrestrial biota by the European Chemical Agency (ECHA) take into account the mortality, the reproduction activity, and morphological and behavioral changes of earthworms, collembola and predatory mites (OECD, 2004, 2016a,b). At the same time, for microorganisms, ECHA evaluates nitrogen and carbon transformation activity (OECD, 2000a, b). In the last years, unexpected negative effects of pesticide residues on non-target organisms were detected in different ecosystems and under different pesticide exposure levels (Desneux et al., 2007; Beketov et al., 2013; Brühl et al., 2013; Wood and Goulson, 2017), and the assumptions of the Regulation Authorities have been often contradicted. In this context, the novel introduction of proper methodologies to ascertain the damage effect of pesticides on the soil living organisms is a decisive step to ascertain any undesired effect at a high tier of safety (Schäfer et al., 2019). Recently, EFSA has issued a scientific opinion on the risk assessment of plant protection products on soil organisms (Ockleford et al., 2017), where microorganisms are also included. While acknowledging the limitations of assays based on single microbial species, EFSA recommends the use of molecular methodologies addressing whole communities but highlights the difficulties of interpreting complex outcomes for regulatory purposes.

Additional endpoints were introduced in the last years as improved estimators of the sub-lethal effects of pesticides, and in this respect, genotoxicity represents a critical marker of xenobiotic exposure having important repercussions not only on the viability of non-target species but also on the ecological fitness of the organisms. Indeed, a clear link between genotoxicity and lowered reproductive performance and embryotoxicity has been highlighted. Therefore, new methodologies have been introduced to study the effects of sub-lethal doses, i.e., doses which have no lethal effects on the organisms, versus soil biota: comet assay, denaturing gradient gel electrophoresis (DGGE), and more recently next-generation sequencing (NGS). The comet assay addresses DNA damage on invertebrates, while DGGE and NGS use DNA and RNA biomarker genes as molecular tools to evaluate changes in the microbial community.

The review describes the researches performed in the last years on the effect of pesticides on soil microorganisms and non-target organisms, using new approaches involving DNA extraction and sequencing (DGGE; NGS) to characterize the microbial population and DNA damage assessment (comet assay) to determine the effect on soil invertebrates.

The aim was to detect the impact of pesticide sub-lethal doses in agricultural soil ecosystems and help European Registration Authorities to derive ecotoxicological parameters useful for the pesticide Registration Procedure at a high tier of risk assessment, taking into account the difficulties of interpreting complex outcomes for regulatory purposes.

## Article Types

Reviews and experimental papers dealing with the effects of pesticides on soil organisms as assessed by means of DGGE, NGS, or comet assay.

## Critical Analysis of the Scientific Literature

### Denaturing Gradient Gel Electrophoresis

Over the last decade, PCR-based molecular fingerprinting techniques, giving a direct comparative overview of the composition and diversity of soil microbiota, replaced most other post-PCR analytical methods (van Elsas and Boersma, 2011). Specific examples of direct molecular monitoring approaches in soil microbiology are represented by DGGE, temperature gradient gel electrophoresis (TGGE) (Heuer and Smalla, 1997; Muyzer and Smalla, 1998), terminal restriction fragment length polymorphism (T-RFLP) (Kuske et al., 2002), single-strand conformational polymorphism (SSCP) (Schwieger and Tebbe, 1998), ribosomal internal spacer analysis (RISA) (Ranjard et al., 2000), and length heterogeneity-PCR (LH-PCR) (Ritchie et al., 2000). Among culture-independent fingerprinting methods, the DGGE, firstly theoretically described in the early 80s by Fischer and Lerman (1979), has been widely applied (Hoshino and Matsumoto, 2007; Coppola et al., 2011; Umar et al., 2017). To obtain optimal results in DGGE analysis, the first practical aspect concerns the high-quality extraction of total DNA from samples and then the control of amplification via PCR selecting universal primers targeting part of the 16S or 18S rRNA sequences for bacteria and fungi, respectively. Subsequently, the separation is based on the differences in mobility of partially melted DNA (with the same length but different sequences) in polyacrylamide gels containing a linear urea and formamide gradient of DNA, properly set (Fakruddin and Mannan, 2013). The identification of the microbial species can be obtained by sequencing the band excised by the polyacrylamide gel (Salles et al., 2004).

Denaturing gradient gel electrophoresis was originally developed to detect small mutation changes in DNA sequences, but quickly the technique has been applied for transversal microbial analyses, thanks to evident advantages such as rapidness, high reproducibility, and low costs (Fakruddin and Mannan, 2013). Currently, PCR-DGGE is a routine and widely used method in soil ecosystem studies. Nevertheless, the technique has some limitations such as PCR biases, variable DNA extraction efficiency, difficult sample handing (Fakruddin and Mannan, 2013), and limited sensitivity (Nocker et al., 2007). Other problems in DGGE analysis could be the formation of heteroduplex molecules, which can alter the distribution of the bands in the acrylamide gel (Ercolini, 2004).

As discussed below, DGGE is also suffering from a very limited resolution power as compared to NGS, with the latter replacing it in several studies. Nevertheless, DGGE is still commonly employed for soil microbial ecology studies in order to monitor population structure and dynamics during that time, especially because of its ability to provide a representative profile of the dominant microbial diversity from specific environments such as soils (Ercolini, 2004; Munaut et al., 2011). In particular, DGGE is a useful tool for the rapid evaluation of microbial profiles in complex ecosystems (Kurtzman et al., 2011; Ng et al., 2014; Di Lenola et al., 2017; Zhang C. et al., 2017), allowing a rapid and efficient separation of the DNA fragments (Umar et al., 2017).

In common with the NGS methods discussed below, different primer sets can be used in PCR-DGGE, thus addressing microbial communities at the phylogenetic level (e.g., 16S primers for bacteria and archaea, ITS for fungi) or at the functional level, depending on functional selected genes. PCR-DGGE protocols optimized for use with soil DNA constitute a consolidated and reliable method that can be used in addition to culture-dependent methods to obtain a complete picture of microbial diversity and dynamics. It can also be as a possible alternative to the most modern NGS techniques since it does not require complex bioinformatics for the analyses of results (Shokralla et al., 2012; da Silva Barros et al., 2019; Hemmat-Jou et al., 2019; Ruanpanun and Nimnoi, 2020).

The analysis of the relevant articles published in the last years on the effects of pesticides upon the soil microorganisms, determined through the DGGE technique, is summarized in Table 1.

**TABLE 1:** Summary of the relevant articles on the application of denaturing gradient gel electrophoresis technique to the pesticide effect on soil microorganisms.

References	Substance	Type	Study	Structure impact	Comments
Hoshino and Matsumoto (2007)	Chloropicrin and 1,3-Dichloropropene	Fumigants	Field	Significant effect	Chloropicrin changed DGGE profiles radically and no recovery was found after 1 year.
Coppola et al. (2011)	Penconazole, Dimethomorph, Metalaxyl, Azoxystrobin, Cyprodinil, and Fludioxonil	Fungicides	Lab	Transitory effects	Evident variation of microbial population after pesticides treatments; no significant differences at the end of the experiment.
Gao et al. (2012)	*Pseudomonas fluorescens* 2P24	Biological control	Field	Transitory effects	Fungal community was significantly shocked at first, but it improved gradually after 1 month.
Gupta et al. (2013)	Chlorpyrifos, endosulfan and azadirachtin	Chemical and natural insecticides	Lab	Dose- dependent significant	High doses of azadirachtin simulated the effects of chemical pesticides
Chen et al. (2013)	*B. subtilis* B579	Biological control	Lab	Minimal and transient effects	Only minimal and temporary changes in rhizobacterial population structure were detected.
Tortella et al. (2013a)	Atrazine	Herbicide	Lab	Transitory effects	Robustness of microbial community toward the treatments.
Tortella et al. (2013b)	Carbendazim	Fungicide	Lab	Transitory effects	Microbial population remained stable over the time when compared to the untreated control.
Lin et al. (2016)	Pentachlorophenol	Herbicide	Lab	Positive effects vs. earthworms	The microbial population was changed by the earthworm treatments.
Huang et al. (2016)	Chlorpyrifos	Insecticides	Lab	Significant effects	The insecticide inhibits the fungal abundance significantly.
Diez et al. (2017)	Atrazine, chlorpyrifos, and iprodione	Herbicide, insecticide, and fungicide	Lab	Transitory effects	Evident variation of microbial community detected only at first treatment.
Wang et al. (2019)	Metalaxyl	Fungicides	Field	Significant effects	Metalaxyl inhibits the growth of fungi.

Recently, Di Lenola et al. (2017) compared different molecular methods to assess soil microbial diversity considered as a complex habitat. Delgado-Baquerizo et al. (2016) already established that anthropogenic pressures such as chemical pollution or agriculture practices strongly affect microbial composition and identified that PCR-DGGE as a rapid method to highlight this shift is promising.

Pesticides are defined as bioactive and toxic substances that can influence, directly or indirectly, soil productivity, and agroecosystem quality (Joergensen and Emmerling, 2006). Their principal function is the growth inhibition of target organisms even if their effects can be extended to non-target microorganisms, causing an alteration in the microbial community structure (Tortella et al., 2013a, b). The evaluation of pesticide impact on non-target organisms in soils, including microorganisms, could be a useful tool to monitor soil health and evaluating their quality. Some years ago, Imfeld and Vuilleumier (2012) already made an extensive investigation on industrially produced pesticides in agriculture in relation to the contamination of soil ecosystems. The authors considered a large variety of cultivation-dependent and -independent methods potentially applied to measure and interpret the effects of pesticide exposure, expanding the study in the specific context of the responses of the soil microflora to pesticide exposure. They established a systematic combination of microbial culture-based and molecular culture-independent methods that will comprehensive contribute to this field.

Gao et al. (2012) compared DGGE and T-RFLP techniques for monitoring the effect of a natural pesticide, *Pseudomonas fluorescens* 2P24, on the soil fungal community in the cucumber rhizosphere. In this case, DGGE results indicated that the fungal community was shocked at the beginning of the trial, but it improved gradually after 1 month, with the decline of *P. fluorescens* 2P24. This study revealed the transient effect of biological control agents against the microbial populations.

Analogously, Chen et al. (2013) studied the effect of *Bacillus subtilis* B579, another natural biological pesticide, on rhizobacteria community structure, using cultivation-based analysis coupled with DGGE to profile the changes of bacterial community structure. As expected, also in this case, the analysis revealed a minimal and transitory effect on microbial populations.

Gupta et al. (2013) applied a DGGE approach to compare the effects of two chemical pesticides (chlorpyrifos and endosulfan) and a biopesticide (azadirachtin) on the bacterial community in rhizospheric soil. In this case, results showed that high doses of azadirachtin simulated the effects of chemical pesticides on bacterial communities showing a significant dose-dependent effect.

The efficacy of natural biopesticides emphasizes the need to widely investigate their effect in agriculture before accepting them as safe alternatives to chemical pesticides. Tortella et al. (2013a; 2013b), studied the effects of chemical pesticides (atrazine and carbendazim) on the microbial community of a biopurification system, revealing a transient reduction of the microbial population after each treatment. Indeed, the DGGE analysis confirmed that microbial structure remained stable for a long time.

More recently, Diez et al. (2017) evaluated the role of the rhizosphere in pesticide dissipation and consequential microbial community changes in a biopurification system. Analogous to the results obtained by Coppola et al. (2011) and Marinozzi et al. (2013), they found that microbial communities were immediately modified 1 day after the fungicides treatments, but the community structure was recovered at the end of the experiment. The shift in the composition was thus only transitory and, at the end of the trial the populations, returned to their initial robustness.

Umar et al. (2017) recently applied DGGE fingerprinting of 16S rDNA to study the bacterial profile of Nigerian agricultural soil. This study confirmed the complex nature of this matrix, and the PCR-DGGE approach gave the advantage of not requiring previous knowledge on this habitat, providing an immediate picture of specific microbial population constituents in both a qualitative and a semi-quantitative way.

In contrast with these critically discussed results, other researchers demonstrate the irreversible effect of chemical pesticides on non-target microbial soil populations. Among them, Hoshino and Matsumoto (2007) revealed that the fumigants chloropicrin and 1,3-dichloropropene (1,3-D) have an adverse effect on fungal community structure. They showed that the chloropicrin treatment changed soil DGGE profiles significantly after 2 months of treatment. The DGGE profiles were not completely recovered even after a period of 12 months. On the other hand, the DGGE profiles of 1,3-D-treated soils showed a small change after 2 months of fumigation, but then after 6 months the treated DGGE profiles became indistinguishable from the controls.

The effects of chlorpyrifos on fungal abundance and community structure as revealed by DGGE were assayed by Huang et al. (2016). A significant inhibition of fungal abundance was induced by chlorpyrifos, a persistent insecticide that is widely used in agriculture despite being very potentially dangerous to non-target environmental organisms. Analogously, Chu et al. (2008) observed the inhibitory effect on the fungal community by chlorpyrifos.

Wang et al. (2019) studied the ecological toxicity of metalaxyl applications on soil microorganisms; the study of T-RFLP and DGGE revealed that metalaxyl inhibits the growth of fungi.

Lin et al. (2016) studied the impact on the soil microbial community and enzyme activity of two earthworm species during the bioremediation of pentachlorophenol-contaminated soils, evaluating the community structure on day 42 by DGGE with the 16S rRNA amplification of the V3 region. This study addressed the roles and mechanisms by which two earthworm species (epigeic *Eisenia fetida* and endogeic *Amynthas robustus*) affect the soil microbial community and enzyme activity during the bioremediation of PCP-contaminated soils. The results obtained confirmed that the soil microbial community was changed by the earthworm treatments.

Considering the overall critical discussion on microbial soil monitoring in the function of pesticide treatments, we can confirm the usefulness of PCR-DGGE as a screening technique to assess the genetic diversity of microbial communities. Nowadays, DGGE remains a preliminary technique to fast highlight the main differences in microbial community composition and is able to give further information if coupled with culture-dependent microbiological approaches, while thorough assessments can be gained by high-throughput techniques such as NGS.

### Next-Generation Sequencing

Next-generation sequencing is an advanced sequencing method that allows the high-throughput nucleotide sequencing of millions of DNA strands in parallel, resulting in reads that are analyzed through bioinformatics, thanks to the availability of reference sequence libraries. Among the available NGS technologies, Illumina is nowadays mostly applied because of throughput, data quality, and costs per sequenced nucleotide. The use of NGS in recent years has increased in many scientific fields (e.g., Clinical Research, Food Science, Agricultural Science, Toxicology) as it became cheaper and therefore accessible to most labs all over the world (Beedanagari and John, 2014; Rockmann et al., 2019; Wiedmann and Carroll, 2019). The technology provides a thorough depiction of soil microbiology, paving the way for the discovery of previously unexplored compositions and diversities of both culturable and unculturable soil microorganisms (Simon and Daniel, 2011; Thompson et al., 2017). Current approaches used to study the diversity of soil microorganisms by NGS are based on phylogenetic and functional marker genes to address the two main questions: “who is there?” and “what are they doing?” This PCR-based approach often uses the amplification of targeted genomic regions of the 16S rRNA marker gene for bacterial diversity and 18S or ITS (internal transcribed spacer) for fungal diversity to answer the first question of “who is there?”(Vasileiadis et al., 2013). Despite the fact that the method itself is highly sensitive and is often the representative of microbial diversity, the presence of relic DNA (DNA of extracellular origin/from the cells that are no longer intact) is its main drawback (Carini et al., 2016). Moreover, the use of metagenomic analysis in combination with other methods (e.g., metatranscriptomics, metabolomics) to reveal the impact of active members on the results thus answering the latter question of “what are they doing?” has been recently adopted by many (Jansson and Baker, 2016; Jansson and Hofmockel, 2018; Shakya et al., 2019). The method is thus a useful approach to understand the impacts of pesticides on soil microorganisms from rhizosphere to bulk soil, both in the short and long term. A review by Imfeld and Vuilleumier (2012) suggested that up until that year of publication, the use of NGS to investigate the impact of pesticides on the soil bacterial populations has not been reported. However, our current review shows that it is almost of common use nowadays, making precedent approaches almost obsolete, mainly because of its unprecedented sensitivity. Indeed, NGS, besides revealing the effectiveness of the applied pesticide on its target, may also reveal the unintended impact of pesticide through screening of non-target groups present in soil (e.g., impact of insecticide on bacteria, herbicide, on fungi) (Kollah et al., 2019; Mallet et al., 2019). In addition, it also has the potential to reveal crucial information regarding the presence and activity of microbial degraders of pesticides and other contaminants in the soil for remediation purposes (Jeffries et al., 2018).

The studies evaluated in this review include works that were focusing on the impact of various types of pesticides (i.e., herbicides, fungicides, insecticides, fumigants, nematicide, and one novel bacteriocide) either separately on bacteria and fungi or on bacteria and fungi altogether. Interestingly, as shown in Table 2, most of the works focused only on the impact of one to three pesticides on either bacterial or fungal communities. Present literature review shows that, while some of the earlier works investigated biodiversity of soil microbial communities often focusing on commonly used regions of the DNA, some of the most recent works, on the other hand, focused on specific communities focusing on various roles and specific genes to correctly evaluate the impact of the treatments on the functionality of soil fertility’s key players (Gallego et al., 2019; Tang et al., 2019; Fang et al., 2020). Only two studies, Armalyte et al. (2019) and Panelli et al. (2017), focused on the overall impact of the specialized farming systems and their relative pesticides’ overall impact on either bacterial or fungal communities.

**TABLE 2:** Summary of the relevant articles on the application of next-generation sequencing to the pesticide effect on soil microorganisms.

References	Substance	Type	Study	Comments
Wang et al. (2020)	Azoxystrobin	Fungicide	Lab	Significant dose dependent impact of the substance on the bacterial community diversity and changes in its composition following exposure to specialized degraders.
Farthing et al. (2020)	Glyphosate and imidazolinone	Herbicide	Field	Transient significant and overall little impact following treatments. Changes mostly attributed to the field properties.
Fang et al. (2020)	Chloropicrin, dazomet, dimethyl disulfide, allyl isothiocyanate and 1,3-dichloropropene	Fumigant	Lab	Transient significant changes with initial diversity decline and brief stimulation. Fumigant type dependent various responses from soil bacterial community and its denitrifiers.
Zhang D. L. et al. (2019)	1,3-dichloropropene	Fumigant	Field	Bacterial community composition remained unaffected by 1,3-D fumigation whereas its impact was detrimental to biodiversity of bacteria, AOA-amoA and AOB-amoA genes.
Zhang C. et al. (2019)	Pyraclostrobin	Fungicide	Lab	Significant impact in the abundances of genera with important roles in soil fertility and pollutant biodegradation.
Tang et al. (2019)	Glufosinate (glyphosate)	Herbicide	Lab	Some variations in bacterial diversity of rhizosphere caused only by plant growth stages. These changes were not attributed to treatments.
Mallet et al. (2019)	Leptospermone	Herbicide	Lab	Significant shifts in community structure and diversity in fungal communities of soils caused by natural weed killer leptospermone. Recovery was only possible for the soil in which indigenous fungal community prior to experiments was already diverse and rich.
Hu et al. (2019)	Fomesafen	Herbicide	Field	A dose dependent effect impact on diversity. Long-lasting significant impact on the soil microbial community and changes toward specialized microorganisms that are able to degrade Fomesafen.
Gallego et al. (2019)	Oxamyl	Nematicide	Field	Increased abundance of the specialized fraction and transient changes in the composition total bacterial community.
Feng et al. (2019)	Lindane	Insecticide	Field	Bacteria were than fungi but stable community structure exhibited in the hybrid rice under lindane stress.
Storck et al. (2018)	Chlorpyrifos, isoproturon, and tebuconazole	Insecticide; herbicide; fungicide	Field	Diversity and composition varied over time more in mesocosms than field. Overall, all pesticides referred as low-risk.
Jeffries et al. (2018)	Chlorpyrifos	Insecticide	Field	Legacy effect after 13 years, community that is able to adapt and degrade OP is still reflected.
Zhang S. T. et al. (2017)	Chloropicrin	Fumigant	Field	Richness and diversity after 3 years continuous fumigation were the lowest. Increase of fumigation years reduced the incidence of bacterial wilt.
Jiang et al. (2017)	Acetochlor (2-chloro-N-(ethoxymethyl)-N-(2-ethyl-6-methylphenyl) acetomide)	Herbicide	Field	No significant impact on soil microbial community composition after 9 years of treatments. Changes between 8th and 9th year were found to be not related to herbicide but to a seasonality.
Chen et al. (2017)	Paichongding neonicotinoid	Insecticide	Lab	Significant, both positive and negative, soil type dependent impact on soil bacterial community. Diversity was found to be gradually increasing in control group while it was decreasing in the IPP group. The inoculation of an IPP degrading strain positively affected the microbial species diversity in contaminated soil.
Panelli et al. (2017)	Farming systems analysis	Various	Field	Fungal and bacterial community alterations caused by various treatments, exact comparison of two systems were not possible.

Particularly, in the work of Armalyte et al. (2019), the comparison of soil bacterial communities revealed no major differences among the main phyla of bacteria between the two farming systems with similar soil structure and pH, suggesting the important role played by these factors. However, slight differences and minor shifts of lower taxa were observed following the treatments and fertilization regimes, and these were interpreted as minor shifts in which the soil community is responding and adjusting itself to handle these treatments and re-stabilize its balance. The study of Panelli et al. (2017), on the other hand, revealed the alteration of fungal consortia starting from the first year of the study, due to various management conditions. It was found that Ascomycota always predominated, with the exception of conventional farming in which high abundance of Basidiomycete species was detected.

The field study of Storck et al. (2018) was the only one in which an insecticide, a herbicide, and a fungicide (namely chlorpyrifos, isoproturon, and tebuconazole) were evaluated altogether. The authors found that the α-diversity of soil bacteria varied more in microcosms as compared to fields. In the field conditions, significant differences in OTUs (operational taxonomic units) were observed for all pesticides by the end of a 70-day-long experiment. Pesticides, regardless of doses implemented, failed to have an impact on the α-diversity indices analyzed in this study. Similarly, also β-diversity was not affected by any of the pesticides nor the doses as the composition of soil bacterial community did not change significantly over time. However, the insecticide chlorpyrifos caused a slight but significant temporary impact at the microcosm level, whereas the fungicide tebuconazole caused a slight but significant temporary impact at the field level only. The herbicide isoproturon, on the other hand, did not cause significant changes in the β-diversity of soil bacteria neither in microcosms nor in the field. These small and transient changes were mainly attributed to various degradation dynamics and transformation products of pesticides. However, as the β-diversity of soil bacteria eventually recovered, all of the pesticides were conclusively referred to as low-risk pesticides.

#### Fungicides

Wang et al. (2020) assessed the impact of the fungicide azoxystrobin on soil microbial communities, revealing a significant dose-dependent impact. Particularly, the OTUs richness and biodiversity, according to the Shannon index, decreased. Furthermore, *Streptomyces* and *Sphingomonas* were found to be dominating genera in most of the treated soils. This particular result was attributed to the use of the pesticide as a carbon source for growth because both *Streptomyces* and *Sphingomonas* hold a potential as bioremediation agents in the soils contaminated with pesticides. Furthermore, in a study carried out by Zhang C. et al. (2019), it was found that a commonly used fungicide pyraclostrobin also caused a significant impact in the abundances of genera with important roles in soil fertility and pollutant biodegradation. However, this time in contrast to azoxystrobin, the fungicide pyraclostrobin decreased the relative abundance of the *Sphingomonas* genus regardless of exposure levels. *Cupriavidus*, *Methylobacillus*, and *Methylophilus*, genera that include degraders of pollutants and methane oxidators with some roles in salt stress tolerance and denitrification were also affected negatively.

#### Insecticides

Feng et al. (2019) studied the impact of lindane on root-associated microbiomes of rice and found that root-associated bacteria were more sensitive to the presence of this insecticide as compared to fungi. The α-diversity analyzed through the Shannon index revealed that the higher insecticide levels had significantly decreased the bacterial and fungal diversity of both rhizosphere and bulk soils. It was also found that regardless of rice cultivars used, lindane significantly decreased the α-diversity also in the endosphere. Furthermore, their results showed that bacteria and fungi were both affected by lindane, respectively, in the phylum and class levels. Chloroflexi, Proteobacteria, and Actinobacteria were found to be dominant phyla, while contrasting responses to lindane from Acidobacteria and Firmicutes were obtained. In the case of fungal community, Basidiomycota and Ascomycota were found to be dominant phyla with several classes of both dominating most of the rhizosphere. However, lindane at various doses did not significantly change the composition of the fungal community.

The longest-term field study considered in this review was that of Jeffries et al. (2018), in which the legacy effect of the Chlorpyrifos insecticide was revealed. It was found that the degradation of pesticide was maintained even after its discontinued use 13 years ago, and the soil microbial community was able to adapt and degrade the pesticide but was still reflected in the latest samplings. Authors found that α-diversity was negatively related to degradation rates, which explained the slower degradation of chlorpyrifos in highly diverse soils. Microbial abundance, on the other hand, together with metabolic function abundances, was found to be positively related to degradation. Results related to a higher abundance of both microbes and pathway mechanisms employed by these microbes were further explained by taxonomic analysis and degradation assays.

Furthermore, Chen et al. (2017) investigated the impact of a novel neonicotinoid Paichongding (IPP) on soil bacterial community in a study in which pyrosequencing was used as one of the early studies of NGS in the present review. Degradation of IPP and its four variants were also investigated. A significant impact of IPP in this study on soil bacterial community, both positive and negative, was found to be closely connected to soil type. During the course of the experiment, α-diversity was found to be gradually increasing in the control group while it was decreasing in the IPP group. This contrast eventually caused significant differences between control and IPP-treated soils. Chen et al. (2017) further investigated the impact of the inoculation of an IPP degrading strain into soil in their experiment and found that the inclusion of IPP degrading strain positively affected the microbial species diversity in contaminated soil. Furthermore, IPP also caused significant changes in the composition of the microbial communities after spraying. Differences were found both at the phylum and genus levels in both inoculated and non-inoculated soils due to accumulation of IPP metabolites.

#### Herbicides

Farthing et al. (2020) found that commonly used weed suppression techniques of repeated glyphosate application, repeated glyphosate application + imidazolinone herbicide use, and repeated glyphosate application + mechanical above-ground biomass removal had only little impact on bacteria and archaea. In particular, none of the these weed-suppressing techniques had a significant impact over controls in terms of α-diversity indexes (species richness and Shannon diversity). The only significant difference was observed between communities of different locations, but this difference was found to be related only to the field differences rather than treatment impact. Although overall changes and compositions generally suggested the absence of overlap following treatments with herbicides, Farthing et al. (2020) found that changes in a number of OTUs were quite similar regardless of the experimental site. This phenomenon was still observed a year following the treatment. The authors also highlighted the necessity of different field site inclusions in the trials, as various responses eventually led to minimal changes that were attributed to pre-existing local microbial communities of soils in these fields.

Tang et al. (2019) investigated the use of glyphosate in the cultivation of transgenic herbicide-resistant plants and found that there was no significant impact on the diversity and structures of rhizosphere bacterial communities in one growing season. The differences in rhizosphere bacteria were only caused by plant growth stages. These results indicated that the growth stage was the most important factor influencing rhizosphere bacterial communities’ diversity, in contrast to the hypothesized influence of cultivar and herbicide application.

Mallet et al. (2019) studied the impact of leptospermone, a natural b-triketone weed killer, on the fungal community in a microcosms study. It was found that leptospermone caused significant shifts in community structure and diversity in fungal communities of soils used in their experiment. Starting from the beginning of the experiments, significant differences were found in the α-diversity of the fungal community according to Chao1, Shannon, and Simpson between soil types and controls. During the experiments, the impact of herbicide treatment resulted in further significant differences both between soil types and controls. Authors found significantly decreased observed richness even after only 4 days together with changes in fungal community composition in one of the soils used. Even though the impact of the herbicide on α-diversity was different on different soils used in this experiment, fungal community β-diversity changed regardless of soil type. Mallet et al. (2019) also showed that even after the complete degradation of the used herbicide, the recovery of the fungal community was possible. However, recovery was only possible for the soil in which indigenous fungal community prior to experiments was already diverse and rich. The findings indicate how important it is to assess the impact of the various soil types not only from the physicochemical point of view but also from the indigenous community point of view for their resilience.

Fomesafen, a diphenyl ether broadleaf weed killer widely used in soybeans and other legumes, was tested for its impact on the microbial community composition of rhizosphere bacteria by Hu et al. (2019). Depending on the dose of its application, fomesafen had an impact on both α and β diversity. Particularly for α-diversity, according to Shannon index, negative impacts on rhizosphere bacteria were proportional to the application dose, regardless of sampling times. This impact was related to the direct toxicity of fomesafen and competitive changes adapted by some taxa in community. The authors concluded that functional impacts were long-lasting on the soil microbial community despite the rapid degradation rate of this herbicide in the rhizosphere. Finally, Jiang et al. (2017) revealed that the long-term application of acetochlor (2-chloro-N-(ethoxymethyl)-N-(2-ethyl-6-methylphenyl) acetomide) had no significant impact on overall soil microbial community composition after 9 years of treatment. Particularly for α-diversity, observed species, the Chao1, Shannon, and Simpson indexes indicated various alternation between manual weeding and pesticides. This result eventually interpreted as a reduction on the biodiversity of soil bacteria caused by manual weeding was more significant than the one caused by herbicide application.

#### Fumigants

The impact of fumigants chloropicrin (CP), dazomet (DZ), dimethyl disulfide (DMDS), allyl isothiocyanate (AITC), and 1,3-D on the total bacterial community and on denitrifiers were studied by Fang et al. (2020). According to the diversity indexes of Shannon, Chao1, and ACE, the α-diversity of soil bacteria significantly decreased for the whole experiment following the fumigation with CP. However, 24 days after fumigation, diversity in the soils of the remaining fumigants were found to be significantly higher than the controls. Later, sampling revealed that biodiversity of the bacterial community in the soils of DZ, DMDS, AITC, and 1,3-D eventually were not significantly different from controls. The stimulation effect of this fumigant, therefore, remained only as a short-term and transient one. Fang et al. (2020) also found that although initially affected negatively by fumigation, denitrifiers eventually recovered. Relative abundances eventually increased significantly in relation with stimulated microbial denitrification caused by fumigation of soil.

Zhang D. L. et al. (2019) also investigated the impact of 1,3-D fumigation on soil bacterial community. The authors also paid particular attention to the abundance of ammonia oxidation genes of the bacterial community in an attempt to evaluate again the unintended impact that a fumigant could have on soil fertility. It was found that soil fumigation with 1,3-D reduced the abundance of total bacteria and AOA-amoA and AOB-amoA genes. It was found that 1,3-D fumigation significantly reduced the total number of species according to the Ace and Chao diversity indexes. The bacterial community recovered from this reduction as the experiment continued.

Finally, in a multi-year field study by Zhang S. T. et al. (2017), the impact of chloropicrin fumigation on the bacterial community of soil was investigated for a 3-year-long continuous fumigation. Continuous fumigation for 3 consecutive years was found to be detrimental for the soil bacterial community in terms of microbial richness and diversity. In detail, α-diversity, as measured by the Chao and Shannon Index, was found to be significantly lower after 3 years of fumigation when compared to control and year-long fumigation. Although slightly to a lesser extent than 3 years, fumigation after 1 year was still detrimental to both microbiota and species richness. Differences were further investigated in the phyla and genus levels to evaluate the impact the duration of fumigation had on the bacterial community. Differences between a year-long and 3-year-long fumigation significantly increased only at the genus level. Authors further implemented the least discriminant effect size analysis to be able to identify the main phylotypes behind the differences obtained. It was found that Nitrospirae and Saccharibacteria were two most prominent phyla in, respectively, no-fumigation and 3-year-long fumigation samples. However, identifying a biomarker for a year-long fumigation at the phylum level was not possible. Therefore, the authors identified *Nocardiopsis* at the genus level as a biomarker for a year-long fumigation sample. Authors also found that increased fumigation years reduced the incidences of bacterial wilt disease.

#### Nematicides

The study of Gallego et al. (2019) was the only one dealing with the impact of a nematicide on soil bacteria: the molecule studied was oxamyl, and particular attention was also devoted to degradation kinetics. Soil bacterial community was not significantly affected by oxamyl in terms of α- and β-diversity. However, the abundance of a specialized fraction of oxamyl degraders increased in agreement with mineralization of the nematicide. The nematicide use also increased the abundance of the specialized fraction of the soil bacterial community carrying the *cehA* gene, which means that oxamyl induced changes in the abundance of oxamyl-degrading microorganisms.

### Comet Assay

Detection of DNA damage and its extent is of paramount importance in different fields of basic and applied medical and health sciences, including environmental studies for verifying the toxicity of xenobiotics or other chemicals released in the environment. Among the different methods developed to quantify DNA damage, the single-cell electrophoresis or comet assay is prominent, being a simple, rapid, and sensitive method for measuring DNA breaks in small numbers of cells (Glei et al., 2016). When standardized and validated, the comet assay can provide valuable information for hazard identification and risk assessment of environmental exposure to environmental pollution in humans, in sentinel organisms, or *in vitro* toxicity studies (de Lapuente et al., 2015).

The method was originally developed in the 80s by Ostling and Johanson (1984) who used the technique to quantify DNA damage in mammalian cells exposed to gamma rays. The technique was further improved by Singh et al. (1988), who developed an alkaline version of the assay, the most commonly used, that enables the detection of alkali labile sites in addition to single- and double-strand breaks visualized using the original neutral version. The assay involves very limited processing steps, not requiring DNA isolation and purification. On the contrary, cells are directly embedded in a low melting agarose matrix at temperatures compatible with cellular viability and stratified on a microscopy slide. After gel solidification microgels on slides are subjected to a lysis step in a saline solution containing detergents that promote cellular membrane degradation and protein precipitation leaving on the slide nucleoids constituted simply by DNA. Subsequently, slides are transferred into an electrophoretic chamber, and DNA is allowed to unwind in electrophoresis buffer, that in the version developed by Singh is at pH > 13, and electrophoresed in the same buffer. Finally, cells are neutralized and either stained with DNA intercalating dyes, mainly fluorescent such as DAPI, ethidium bromide or SYBR Gold and analyzed directly or dehydrated for further analysis. Analysis is conducted using a fluorescent microscope; the image of the comet produced by electrophoresis is represented by a head of intact DNA and a tail of damaged DNA streaming away from it in the direction of electrophoresis. Classification of comet according to their DNA damage can be either done using visual scores but more commonly is supported by an image analysis software that is capable of extrapolating numerical indexes of damage, the most common of them being the length (Tail Length) of the comet and the percentage of DNA present in the comet (Tail Intensity%). A third index of reference widely used is a product of the previously mentioned indexes and is known as Tail Moment. New promising techniques in the classification of comet cells is provided by artificial intelligence algorithms applied to image analysis that will improve and accelerate the classification process in the near future (Bernardini et al., 2019). Despite its popularity, the comet assay still has some shortcomings mainly due to a high inter-operator as well as inter-laboratory variability and the limited use of calibrators. Indeed, although the process is very straightforward, limited variations in each one of the methodological steps described may complicate the comparison of data from different laboratories, in particular when the starting material is not constituted by cultured cell lines as in the case of reference organisms used in the ecotoxicological assessment. One of the purposes of the present review is to summarize not only the application but also some critical methodological details in order to describe the most suitable methodological approaches in line with the paper published in the past 5 years in the field of soil environmental ecotoxicology.

The analysis of the relevant articles published in the last years on the effects of pesticides upon the soil earthworms, determined through comet assay, is summarized in Table 3.

**TABLE 3:** Summary of the relevant articles on the application of comet assay to the pesticide effect on soil earthworms.

References	Substance	Type	Study	Organism	Comment
Ma et al. (2019)	Pyraclostrobin	Fungicide	Lab	*E. fetida*	DNA damage increases at 7 and 14 days.
Espinosa-Reyes et al. (2019)	Persistent organic pollutants	“Pesticide”	Field	Not specified	DNA damage correlated to POP accumulation in soil.
Qiao et al. (2019)	Cyantraniliprole	Insecticide	Lab	*E. fetida*	Increase oxidative stress damage alters antioxidant cellular status inducing DNA damage.
Zhang et al. (2018)	Fluoxastrobin	Fungicide	Lab	*E. fetida*	Oxidative stress parameters correlated to DNA damage.
Li B. et al. (2018)	Acetamiprid	Insecticide	Lab	*E. fetida*	Maximum of DNA damage after 14 days. Recovery phase started after 21 days.
Chen et al. (2018)	Tribenuron methyl Tebuconazole	Herbicide and fungicide	Lab	*E. fetida*	Not significance changes in OTM and TL at all pesticides concentrations alone and combined.
Li X. et al. (2018)	Mesotrione	Herbicide	Lab	*E. fetida*	Maximal OTM values at highest dose at day 28.
Chevillot et al. (2017)	Neonicotionoids	Insecticide	Lab	*E. Andrei*	The low NEOs concentrations were not lethal but induce significant increase in class 4 (extremely DNA damage).
Duan et al. (2017)	Polychlorinated biphenyls	Pesticide	Lab	*E. fetida*	PCB treatment increase DNA damage in both type of soil, even at lowest concentration tested.
Wang et al. (2016)	Imidacloprid	Insecticide	Lab	*E. fetida*	OTM and tail DNA% increased at 7 days increasing doses. At 21 and 28 days DNA cross- links were observed.
Fouché et al. (2016)	Biofumigants, glucosinolates	Natural toxins	Lab	*E. Andrei*	Broccoli and oilseed radish DNA damage level similar to ctrl. Only Mustard significantly different to ctrl.
Shen et al. (2015)	Deltamethrin	Pesticide	Lab	*E. fetida*	Nickel produces more DNA damage compared to Deltamethrin. Synergistic effect in increasing DNA damage.
Zhang et al. (2015)	Spirotetramat	Insecticide	Lab	*E. fetida*	Extent of DNA damage reveal 90% worms have low DNA damage at 0.25 mg/kg.
Feng et al. (2015)	Thiacloprid	Insecticide	Lab	*E. fetida*	1 and 3 mg/kg reached higher DNA damage at 28 and 35 days.
Mincarelli et al. (2019)	Copper sulfide	Pesticide	Lab	E. andrei	DNA damage at concentrations lower than those found in most agricultural soils worldwide after 9 days of exposure.
Wang et al. (2015)	Guadipyr	Insecticide	Lab	*E. fetida*	Guadipyr has no effect on OTM, TM, Tail DNA%.

Among the techniques developed to assess DNA damage, the comet assay has received remarkable interest for its easy and cost-effective implementation. Although it was originally designed mainly for human and cultured cells, ecotoxicological applications widened the analysis to a broad set of invertebrate species. Within ecotoxicological applications, the technique was primarily used for genotoxicity assessment in marine and freshwater invertebrates, and subsequently, it was extended to terrestrial invertebrates. Soil invertebrates are known to be efficient accumulators of xenobiotics and to respond to their exposure in a sensitive and measurable manner, hence their popular use as bioindicators of soil contamination. The comet assay has been applied to various annelids including polychaetes, oligochaetes, and leeches, although the majority of studies were carried out on selected species of earthworms (*Eisenia* spp.) that are recognized models to assess soil quality and environmental impacts of cropping systems and pollutants. In fact, among soil organisms, earthworms deserve particular interest because of their ecological role in soil biocenosis representing 60–80% of the total biomass; by ingesting soil particles, they represent extremely pertinent bio-indicators (Sanchez-Hernandez, 2006).

Sub-lethal markers such as DNA damage are pivotal to identify potential environmental risks, taking into account the chronicity of exposure of non-target organisms to pollutants widely used in agricultural practices (e.g., insecticides, fungicides, and herbicides) (Di Marzio et al., 2005).

The bioavailability of pesticides in soils is affected by the amount and quality of pesticide-absorbing soil colloids and microbial growth and activity, which results in a different extent of pesticide biodegradation (Castillo et al., 2008; Katagi, 2013).

According to the relevance of earthworms in soil ecotoxicology studies, in the present review, we revised the application of the comet assay in the investigation of environmental pesticide sub-lethal effects in these non-target species. The literature of the last 5 years on the topic consists of 16 articles: only one among them was conducted in field conditions related to the effects of persistent organic pollutants (POPs), including those introduced as pesticides in the environment (Espinosa-Reyes et al., 2019). In particular, in this study, genotoxicity was tested in wild earthworms in soils at different levels of urbanization (industrial, urban, and rural areas). The analysis unambiguously identified the highest concentration of POPs and the highest rate of DNA damage in industrial areas. Urban and rural areas had a different composition of POPs with the more prominent influence of agrochemistry in rural areas; however, the latter seemed to have a lower impact, compared to urban organic pollutants in triggering DNA damage in earthworm coelomocytes.

With the exception of this paper, all other screened articles were conducted in standardized laboratory conditions through the application of OECD guidelines. Moreover, these studies did not use wild earthworms; on the contrary, they mainly used as a reference organism *E. fetida*, with the exception of three articles (Fouché et al., 2016; Chevillot et al., 2017; Mincarelli et al., 2019) that analyzed the effects of pesticides on the other commonly used earthworm reference species *Eisenia andrei*. As reported in Table 3, most of the studies used three organisms as an experimental unit for experimental conditions, and exceptionally few studies increased the observational sample to up to 10 or 18 samples (Wang et al., 2015; Chen et al., 2018; Espinosa-Reyes et al., 2019). In all studies, DNA damage assessment was conducted on coelomocytes – hemocytes of the annelids that can be recovered from the coelomic fluid with non-invasive techniques by submerging the organism in ethanol 5% saline solution that might be integrated with the chelating agent EDTA and the mucolytic agent guaiacol or similar. The studies explored the potential genotoxic effect of a broad set of chemicals including as insecticides Cyantraniliprole (Qiao et al., 2019), Acetamiprid (Li B. et al., 2018), Neonicotionoids (Chevillot et al., 2017), Imidacloprid (Wang et al., 2016), Spirotetramat (Zhang et al., 2015), Thiacloprid (Feng et al., 2015), and Guadipyr (Wang et al., 2015) that represented the most numerous, followed by fungicides Pyraclostrobin (Ma et al., 2019), Fluoxastrobin (Zhang et al., 2018), Tebuconazole (Chen et al., 2018), and a smaller set referred to herbicides Tribenuron (Chen et al., 2018) and Mesotrione (Li X. et al., 2018) or general pesticides including Polychlorinated biphenyls (Duan et al., 2017) and natural toxins used as biofumigants (Fouché et al., 2016) and copper sulfide (Mincarelli et al., 2019) that is one of the few chemical treatments allowed in organic farming. In all studies, synthetic compounds were studies at increasing doses of exposure normally below 10 mg/kg of soil, with the exception of the insecticide Guadipyr (Wang et al., 2015) that was used between 10 and 100 mg/kg of soil. Furthermore, typically, DNA damage was evaluated at specific time points, most commonly once a week up to 1 month. Studies by Shen et al. (2015) and Feng et al. (2015) reported in Table 2 investigated the effect of pollutant exposure up to 6 weeks or 2 months. Interestingly in both studies, it was observed that prolonged exposure led to a significant decrease in the rate of DNA damage highlighting a potential adaptation to the environmental stress that is compatible with the ecology of *Eisenia* spp. that is quite resistant and therefore also useful as a sentinel organism for sub-lethal toxicity.

Most of the studies screened used as a reference index Olive Tail Moment (OTM), which is a widely used index of DNA damage that essentially represents the product of the percentage of total DNA in the tail and the distance between the centers of the mass of head and tail regions [Olive moment = (tail mean-head mean) × % of DNA in the tail]. This is quite surprising considering that tail intensity DNA% has been mainly used in recent investigations due to its robustness (Collins, 2004; Kumaravel and Jha, 2006).

DNA damage was often related to markers of oxidative stress that exhibited a similar trend in response to pollutants. This is central in describing the mechanism of genotoxicity of pesticides that often is mediated by an increase in cellular reactive oxygen species formation due to cellular impairment (Zhang et al., 2015, 2018; Duan et al., 2017; Li B. et al., 2018; Li X. et al., 2018; Qiao et al., 2019). Interestingly despite the different nature and target of the pesticides tested, a common pattern of response is often evincible in the biological response of *Eisenia* spp. with a dose-dependent response to the chemical at each time point analyzed; however, when they are observed on a longitudinal time scale during the typical month of exposure (28 days) at the lowest dose of chemicals used, often a decrease in DNA damage is observed from the second week of exposure highlighting an adaptive response; at intermediate dosage, DNA damage tends to stabilize during the last 2 weeks of exposure while at the highest concentration DNA damage is able to increase in comparison to the previous time points even after 1 month of exposure (Li X. et al., 2018; Zhang et al., 2018; Ma et al., 2019). Mincarelli et al. (2019) also showed an adaptive response at the level of gene expression of metallothionein as well as proteins involved in the immunological response (antimicrobial peptide fetidin and toll-like receptors).

The decrease in the extent of DNA damage might be the result of an adaptive response to low doses of chemicals but is also known to be potentially due to the artifactual decrease in the mobility of damaged DNA associated to the DNA-DNA or DNA-protein crosslink. This seemed to be the case of the insecticide Imidacloprid (Wang et al., 2016) that differs from the other pesticides that showed a dose-dependent decrease of DNA damage already after 1 week of exposure despite a significant increase in markers of oxidative stress and damage. Also, the data relative to the genotoxicity of the neonicotinoid insecticide Guadipyr presented by Wang et al. (2015) are in antithesis with other reports showing very low toxicity in general, and also at the DNA integrity level, following exposure at a concentration 10 times higher compared to other pesticides considered in this review. The compatibility of the high doses of chemicals nominally used with the reproduction and viability of earthworms seems to suggest that Guadipyr is remarkably safer compared to other insecticides; however, the lack of positive control of toxicity in the same set of experiments and bioaccumulation data at the organism level might suggest the need for further investigation to better classify the toxicity of this compound.

Finally, a particular mention should be devoted to innovative approaches of the use of the comet assay among the selected articles. Chevillot et al. (2017) studied the biological exposure of earthworms to complex chemical mixture exposure that mimics environmental complex matrixes. Despite the exciting experimental design, however, the section on DNA damage is quite limited and indicates a slight but significant increase in DNA damage of earthworm coelomocytes following exposure to neonicotinoids.

Another application of the comet assay for ecotoxicological soil studies is to investigate the use of particular amendments, such as organic material, and verify whether they could affect the pesticide concentrations and its toxic potential. The study by Shen et al. (2015) developed along this line demonstrating that humic acid alleviated the damage to DNA, proteins, and lipid membranes caused by nickel and deltamethrin spiked in the soil.

Despite the shortcomings mainly associated with operator dependent and interlaboratory variability, the comet assay remains an elective technique for assessing genotoxicity in environmental biomonitoring, being mature after decades of implementation and widely used worldwide for its direct, simple, and affordable implementation. Nonetheless, in order to promote the consistency and reliability of results, regulatory bodies should provide guidelines on the optimal use of this tool, strongly indicating the most reliable indicators of DNA damage. In this respect, most of the articles reported in the review use OTM, while in the last decades, many evidences seem to highlight DNA tail intensity% as the most informative index characterized by a broader dynamic range. Moreover, the use of computer-aided image analysis software rather than visual scoring should be strongly suggested as well as the use of appropriate reference of damage such as negative and positive controls treated with DNA damaging agents or gamma radiation, ideally at least at 2 doses. Reference cells should be used as standard and to verify the reproducibility of the techniques and do not necessarily have to be of the same type of analytical samples; indeed an optimal reproducibility could be achieved with cultured cells.

## Conclusion

The examination of the published articles showed a continuous improvement of the possibility to detect the detrimental effect of the pesticides on soil microorganisms and non-target organisms at a sub-lethal level. In the present review, we considered the methodological aspects considering the most promising DNA-based methodologies used to generate data for biodiversity and biomonitoring studies; moreover, we described the specific outcomes of research papers using these techniques in describing the effect of pesticides on soil. The critical analysis of the papers examined can help registration authorities to derive ecotoxicological parameters useful for a high-tier risk assessment. Regarding microbial communities, soil microorganisms play key roles in significant ecological processes such as bioremediation, recycling of elements, soil structure establishment, and degradation of organic matter and chemical xenobiotics (Umar et al., 2017; Walvekar et al., 2017). Among them, pesticides are the most common contaminants in the agricultural field (Wołejko et al., 2020), and their persistence in the soil can alter the physico-chemical structure and microbiota composition causing a negative impact on soil biodiversity (Diez et al., 2017). Indeed, the extensive use of pesticides has gradually led to soil contamination with proven damages to environmental health (Fernandes et al., 2020; Kafaei et al., 2020). Reported data using DGGE-PCR studies confirmed that the chemical nature and the doses used play a role in the disturbance of microbiological complexity of the soil.

The use of biopesticides, differently from synthetic ones, does not affect the microbial community reversibly (Reali and Fiuza, 2016; Umar et al., 2017). In this respect, several studies have shown that the pesticide-induced microbial changes are mainly transient and the structure of the microbial population can improve gradually (Gao et al., 2012; Chen et al., 2013). However, this advantage is lost at high concentrations. Gupta et al. (2013) found that the treatments of rhizosphere with high doses of biopesticide azadirachtin had an adverse effect on the microbial population. They were suggesting that concerning the environmental impact, the lower toxicity of biopesticide is lost at high concentrations.

Regarding synthetic pesticide impact on microbial soil, some PCR-DGGE studies suggest that also in this case in the long term, they seem not to affect the rhizosphere population negatively. For example, Coppola et al. (2011); Marinozzi et al. (2013), and more recently Diez et al. (2017) show that the acute soil population modification is reversible since over a short time the soil community was able to return to the initial composition as reported by Tortella et al. (2013b).

However, these studies are in contrast with previous evidence (Hoshino and Matsumoto, 2007; Chu et al., 2008; Huang et al., 2016; Wang et al., 2019) showing that synthetic pesticides significantly affect the abundance of soil microorganisms, causing irreparable damage. The evidence shows the ecological toxicity of several pesticides such as metalaxil and chloropicrin on the fungal community (Hoshino and Matsumoto, 2007; Wang et al., 2019).

Further studies using novel high-throughput techniques such as NGS has shown that in laboratory experiments the majority of the pesticides used had a significant impact on the microbial communities with the sole exception of the observations by Tang et al. (2019) in which the changes were attributed to plant growth stages rather than the Glyphosate. In the field experiments, on the other hand, significant changes were mostly depending on the dose, period, and frequency of application, and the characteristics of the soil, including its indigenous microbial community properties. It is therefore not possible to pinpoint exactly one type of pesticide or substrate as the source of major impacts. Several studies indicated that the rapid shift of communities in favor of pesticide-degrading groups already presents in the microbial community. The presence and activity of pesticide-degrading groups before the application of tested pesticides may be considered as preliminary indicators of synergies among molecules and of the possible outcome of pesticide application. Pesticides used had relatively lower impact, as expected, in the experiments where soil microbial communities were already highly diverse and rich from the beginning. Several studies cited in this work underlined the importance of carrying out various samplings throughout the cropping season. This was because changes in microbial composition happen during the growing season via plant–microbes interaction, and some transient changes may also be explained by the presence of the crops rather than pesticides. Furthermore, the replication of the experiments in different fields is also necessary in order to comprehend the real impact of pesticide(s), as different fields have different physical characteristics and microbial diversity. These factors can indeed affect the response and recovery of the microbial communities. Using the comet assay, the reported study analyzed the toxicity of pesticides in non-target organisms, in this case, referring to soil macroinvertebrates, in terms of DNA damage. Remarkably the main effect on soil biocenosis is also confirmed in this studies that support a high level of adaptation and overtime to the pesticides, suggesting that dose and time of observation are critical aspects to take into consideration in environmental risk assessment. Although this aspect may seem quite obvious, this consideration should be addressed in particular in relation to natural compounds that have limited constraint in terms of dosage to be used, whereas the discussed data suggests that their toxicity at high concentrations should be carefully evaluated. In the light of the above cited findings, we can suggest that the way forward for studies on the impact of pesticides on soil ecosystem lies in the inclusion of more than one soil type/field in the studies; samplings at various intervals during the experiment in order to better assess the shifts and recovery of the microbial community; and coupling the overall biodiversity and functionality with that of key microorganisms of agricultural and soil fertility relevance such as denitrifiers, saprophytic fungi, and archaea. The inclusion of these factors into studies would, therefore, pave the way to understanding that also the unintended impact of a secondary nature on the terrestrial ecosystems besides the main purpose of the pesticide should be considered, which is a very important point for both regulatory authorities and researchers. DNA-based methodological tools provide a robust and efficient technique for the evaluation of microbiological biodiversity as well as early biomarkers of genomic damage in soil organisms that represent an early reporter of stress and potential risk for biota survival and reproduction. The major advantage of these techniques is related to the uniformity of protocols and high-throughput capabilities that enable rapid and comprehensive assessment, addressing major challenges in ecological risk assessment (i.e., provide information at higher levels of biological organization moving from individual organisms to communities and population) and the ability to consider multiple stressors and context dependencies, allowing recovery analysis. On the other hand, at the interlaboratory level, care should be taken in order to standardize the methodologies and gain reproducible results using appropriate reference markers. Moreover, information should be handed in an integrated manner with other biological and environmental indicators using multivariate predictive models and multimetric approaches.

## Author Contributions

All authors listed have made a substantial, direct and intellectual contribution to the work, and approved it for publication.

## Conflict of Interest

The authors declare that the research was conducted in the absence of any commercial or financial relationships that could be construed as a potential conflict of interest.
